# Evaluation of Doc’EDS: a French semantic search tool to query health documents from a clinical data warehouse

**DOI:** 10.1186/s12911-022-01762-4

**Published:** 2022-02-08

**Authors:** Thibaut Pressat-Laffouilhère, Pierre Balayé, Badisse Dahamna, Romain Lelong, Kévin Billey, Stéfan J. Darmoni, Julien Grosjean

**Affiliations:** 1 Department of Biomedical Informatics, Rouen University Hospital, Normandy, France; 2 LIMICS U1142 INSERM, Sorbonne Université & Sorbonne Paris Nord, Paris, France; 3 LITIS EA4108, Rouen University, Normandy, France

**Keywords:** Clinical data warehouse, Cohort identification, Electronic health record, Information retrieval, Semantics

## Abstract

**Background:**

Unstructured data from electronic health records represent a wealth of information. Doc’EDS is a pre-screening tool based on textual and semantic analysis. The Doc’EDS system provides a graphic user interface to search documents in French. The aim of this study was to present the Doc’EDS tool and to provide a formal evaluation of its semantic features.

**Methods:**

Doc’EDS is a search tool built on top of the clinical data warehouse developed at Rouen University Hospital. This tool is a multilevel search engine combining structured and unstructured data. It also provides basic analytical features and semantic utilities. A formal evaluation was conducted to measure the impact of Natural Language Processing algorithms.

**Results:**

Approximately 18.1 million narrative documents are stored in Doc’EDS. The formal evaluation was conducted in 5000 clinical concepts that were manually collected. The F-measures of negative concepts and hypothetical concepts were respectively 0.89 and 0.57.

**Conclusion:**

In this formal evaluation, we have shown that Doc’EDS is able to deal with language subtleties to enhance an advanced full text search in French health documents. The Doc’EDS tool is currently used on a daily basis to help researchers to identify patient cohorts thanks to unstructured data.

## Background

In the last 20 years, hospital data collection and storage have increased massively with the widespread use of clinical information systems (CIS). CISs contain large amounts of data on patients’ health and healthcare: billing codes generating diagnosis related groups (DRG), medications, laboratory and imaging results, unstructured data, etc.

Unstructured data embedded in electronic health records (EHR) (mostly narrative reports) are necessary to solve trial eligibility criteria in 59% to 95% of clinical studies [1, 2]. Indeed, a wide range of crucial healthcare data is commonly found within unstructured clinical narratives. Narrative reports allow flexibility of expression such as doubts, negations, or diagnostic hypotheses and complex representation of diseases, clinical examination, patient history, and family medical history [3, 4].

Clinical Data Warehouses (CDWs) enable to register and aggregate different fragmented healthcare data from CISs and secondary data re-use. CDWs can enhance the quality of disease management [5, 6], and support clinical and translational research [7–9]. There are many CDW solutions such as I2B2 [10], STRIDE [11] and Vanderbilt [12] listed in a recent review [13].

Natural Language Processing (NLP) algorithms and information retrieval (IR) in EHR enable clinicians to identify cohorts [14]. In addition, automatic screening for trial eligibility criteria and extraction is currently being developed based on NLP [2, 15].

Mining clinical narrative in full text is already done by EMERSE [16] and CREATE [17] in English and by Dr.Warehouse [18] and eHOP with Roogle [19] in French. However, there is a need to improve the semantic approach to deal with unstructured data: spelling errors, synonyms, abbreviations, acronyms, temporal notions, including all subtle information cited above. Only evaluations of user satisfaction and use cases are provided in the literature, but formal evaluations are lacking.

We present Doc’EDS (EDS = *Entrepôt de Données de Santé* in French, Health Data Warehouse in English), developed by the Department of BioMedical Informatics, Rouen University Hospital, France: a pre-screening tool to search patient profiles, and to identify cohorts in a document-oriented database. The aim of this study was to provide a formal evaluation of the Doc’EDS NLP features (detection of negative, hypothetical and family medical contents and segmentation) that help to deal with unstructured clinical narratives in French.

## Methods

A. Clinical data warehouse

Doc’EDS relies on the CDW developed by Rouen University Hospital in 2018. It contains patient data (birth date, gender) and related clinical data (hospital stays, diagnoses, procedures, unstructured data documents and laboratory results) since 1998.

Doc’EDS is based on a document-oriented database in which each document corresponds to unstructured data documents from the CDW. Related attributes are: patient identity (ID), patient gender, patient birth date, document date, document type (e.g. discharge summary, procedure report), patient age (calculated from the document date and patient birth date), document production unit (e.g. cardiology, urology), DRG diagnoses & procedure codes (ICD-10 & CCAM classifications) and hospital stay ID. CCAM is a French coding system that is used to report medical, surgical, and diagnostic procedures and services to entities such as physicians, health insurance companies and accreditation organizations. A specific ETL (Extract Transform Load) program has been developed to select unstructured data documents and their related attributes from the Rouen CDW via SQL (Structure Query Language) queries.

B. Document processing (NLP features)

**Fig. 1:**
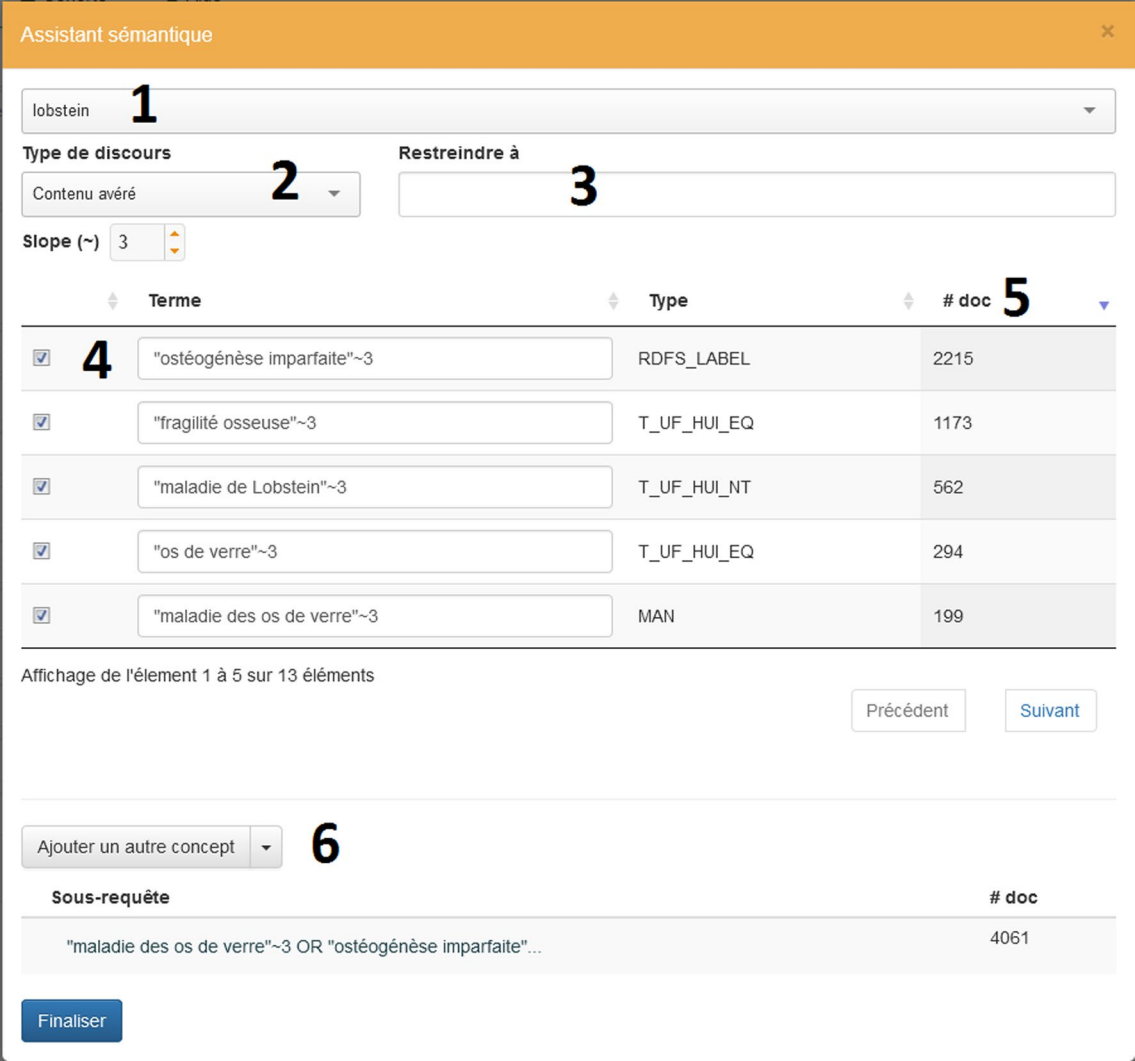
Document processing workflow from extraction to indexing

In order to be integrated into Doc’EDS, clinical narratives are processed through a dedicated workflow (see Fig. 1). First, the documents are extracted from their original database and then converted to a plain text format thanks to the Tika Java library.^1^

*Anonymization* The ETL program has access to the patient entity during the loading operation; it uses the patient’s names (last names & fore names) to remove their occurrences in corresponding texts. In addition, regular expressions are used to ensure that all names and IDs (patient ID but also family ID or physician ID) are de-identified. Patient de-identification was evaluated during the ‘formal’ evaluation “tag and segmentation” detailed below.

*Irrelevant content* In documents, headers and footers contain physician names, medical unit labels but also keywords corresponding to diseases or symptoms managed in medical units (e.g. Alzheimer, Parkinson, headache). This information is ignored in document indexing but remains visible for end users. In order to detect irrelevant content, a global frequency table was created from all documents with line (± 7) and string position. Our methodology to detect irrelevant content (headers/footers) relies on the occurrence of strings/sentences at given positions. The more a sentence is repeated within the different documents, the less relevant it appears. A threshold has been set up arbitrary to 500. Slight differences of line position and useful terms are considered (e.g. *hypertension* which is a common clinical sign). Moreover, some exceptions can be entered into the system (to force or unforce specific strings that should or should not be considered as irrelevant).

*Special content tagging* Doc’EDS is able to detect negative content, hypothetical content and family medical history content. Moreover, when possible, Doc’EDS deals with structured documents with various segments (indication forhospitalization, medical history, etc.). More details are provided about tags and segmentation in the formal evaluation section.

*Document indexing* The ETL program loads documents in a Lucene index: for text search, two distinct fields are created corresponding to patient content and related family content. By default, the patient content is the document text content minus tagged content and comments. Other text fields are generated on the fly by combining segments and “hypothesis” and “negation” sections; for example, it allows to search negative content in specific segments (e.g. NOT *asthma* in anamneses, corresponding to documents in which anamneses contain a sentence which states that the patient does not have asthma). Doc’EDS is implementing the Collector functionalities of Lucene to collect and retrieve results as fast as possible. Each query returns a collection of document IDs and the corresponding number of patients (a single patient can be represented by multiple documents relative to the query).

III. Interface design

**Fig. 2:**
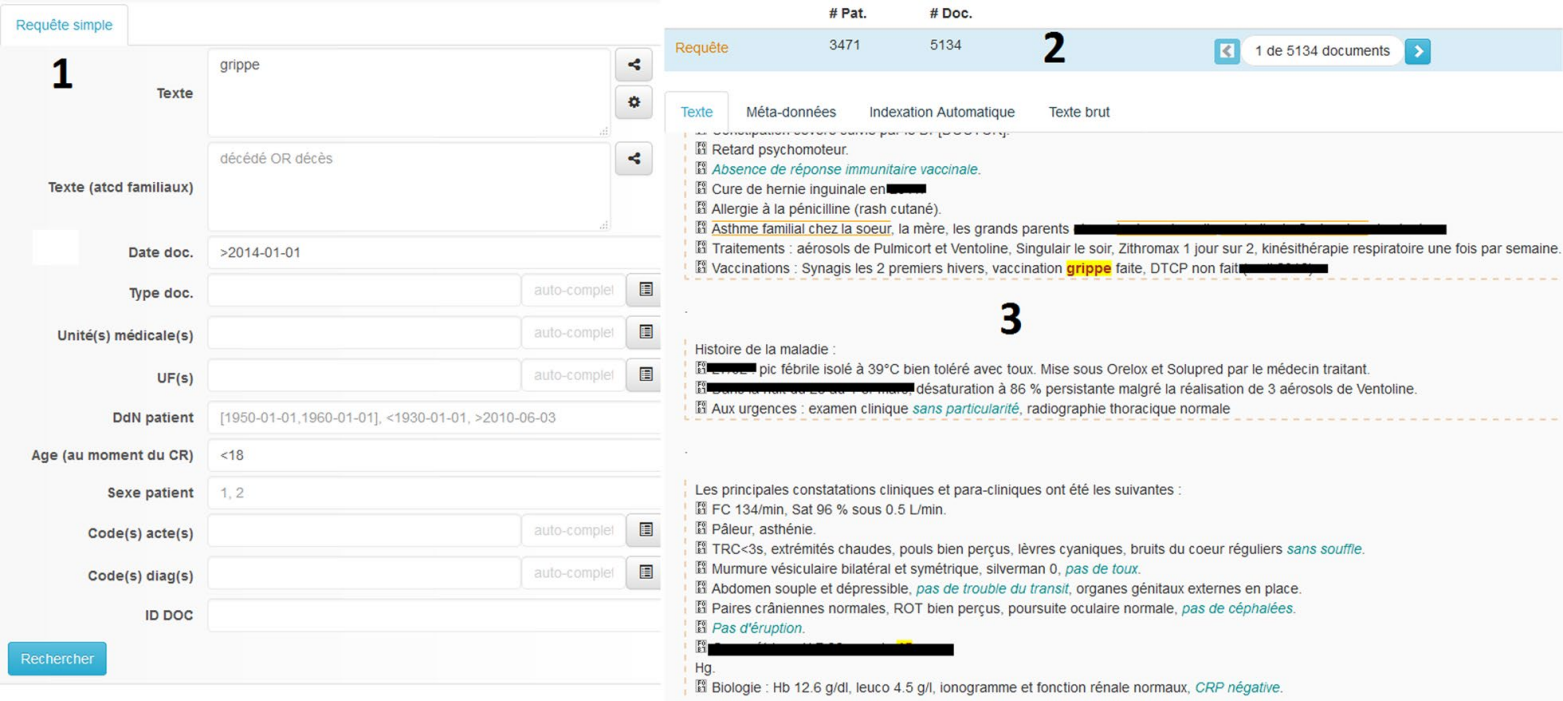
Screenshot of Doc'EDS main page: (1) the query form is on the left side. In addition to keywords, different fields can be used e.g. document date, type, patient age and sex, etc. (2) the number of patients and documents retrieved are displayed, (3) A visualization screen allows users to consult documents (in order to refine queries or collect specific data). In this example, some portions of text (dates) have been blanked to preserve patientanonymity

**Fig. 3:**
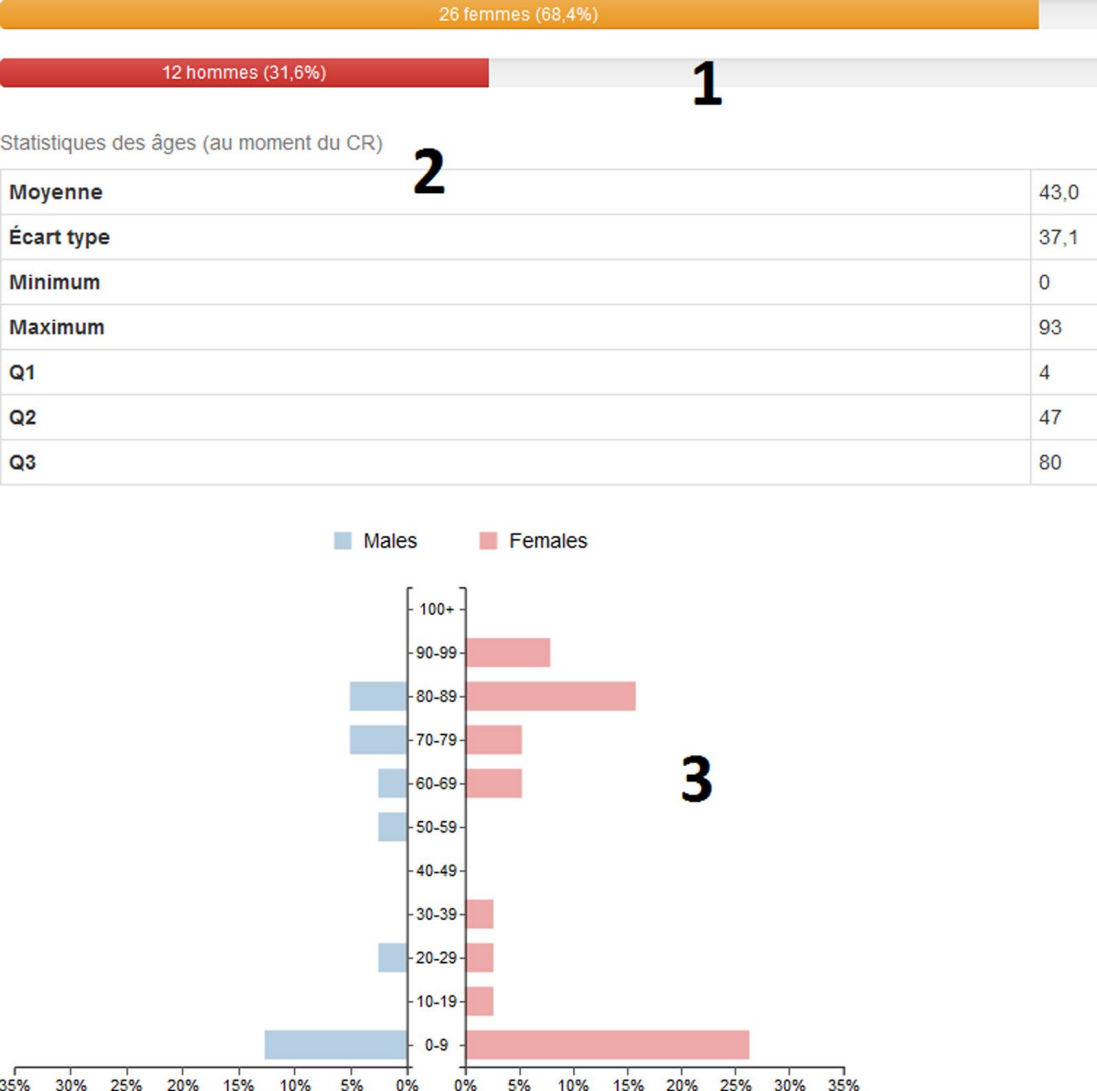
Example of analysis panel function for the query “psychomotor regression” AND epilepsy: (1) sex distribution, (2) descriptive statistics concerning age, (3) age distribution by sex

A screenshot of the main query/visualize window is presented in Fig. 2. The analysis panel is useful to refine queries or to collect important information; Fig. 3 is an example of the age distribution for the query *“psychomotor regression” AND epilepsy*.

IV. Query assistant

### Building queries

Keywords can be entered and enhanced with the following advanced options: *wildcards* (*) can be used to deal with variations in spelling (e.g. pso* for *psoriasique, psoriasis…*); *double quotes* (“”) can be used to search exact word sequences (e.g. *“allergie au paracetamol”*); *slope* ( ~) can be used to take into consideration distance between (non-stop) words (e.g. “*abcès anal”* ~ *2* can have a variation form as *abcès de la marge anale*); *Boolean* operators can be used to combine terms (e.g. *(paludisme OR “accès palustre” OR palu) AND quinine).* A specific module allows to search in one specific segment or to specify if negative or hypothetical clinical concepts are kept in search results or not. Keyword queries can be combined with structured data (age, sex, document type, medical unit, ICD-10 or CCAM codes).

### Query semantic expansion

**Fig. 4:**
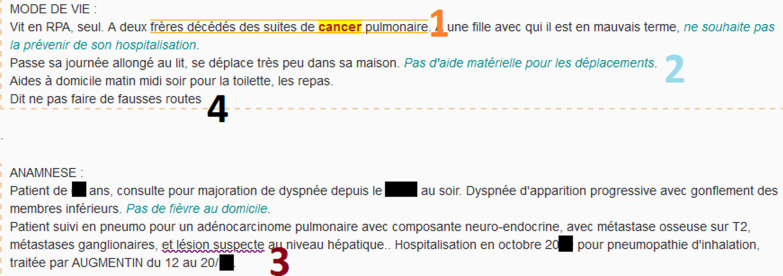
Capture of the semantic assistant that helps the user to enhance the query. 1) Type words to search in HeTOP and select a concept, 2) Select part of speech including negations, hypothetical parts or family history, 3) Select segment(s) to query, 4) Choose relevant terms (which are synonyms or hyponyms extracted from HeTOP) to add to a query, 5) Check the number of documents found in real time for each proposed term and 6) If needed, repeat the operation by adding a new concept (with OR, AND or NOT operators)

Doc’EDS relies on the HeTOP crosslingual multi-terminology server [20] (URL: www.hetop.eu) which contains terminologies and ontologies in 45 languages (mainly in English and in French). Among the 85 termino-ontologies integrated into HeTOP, only 18 are also integrated in the UMLS [21]. Several French terminologies (such as NCIT, SNOMED CT, ICD-O, OMIM, Radlex, FMA, etc.) have been partially translated into French. In September 2020, UMLS contained around 158,475 concepts sharing the same Concept Unique Identifier (CUI) with at least one translation in French whereas HeTOP contains 444,258 concepts with at least one translation in French. Terminologies exist only in French; e.g. CCAM for procedures or BNPC for chemical substances. HeTOP contains over 540,000 health concepts, with a HeTOP Unique Identifier (HUI, which is similar to the UMLS CUI including other non-UMLS terminologies). From these 540,000 HUI, we were able to create a French health lexicon (COMuF), similar to UMLS Specialist Lexicon. The function allows to search synonyms and related terms to leverage the original simple query in order to expand the number of documents (lexical variations, acronyms, etc.). In addition, the number of documents with each term is provided on the fly. This expansion is provided by the HeTOP server that provides a web service to query the CoMUF lexicon. For each concept found, its synonyms, hyponyms and related terms are fetched then automatically used as a subquery to Doc’EDS. When a term matches at least one document, it is proposed in the module to the end user who can choose to modify, add or delete terms dynamically (see Fig. 4, with an example for “osteogenesis imperfecta”).

E. Automatic analysis

The number of documents and patients are displayed for each query. An advanced tool using structured data provides descriptive statistics as aggregated data (tables and charts) from all the retrieved documents: demographic data (pyramid of ages, male/female ratio), lists of ICD-10/CCAM codes, dates/types of documents, and medical units. This tool has two purposes: 1/it can help the user to refine the query (e.g. exclude a specific medical unit from the query) and 2/it can provide direct quantitative information (e.g. what is the median age of patients who had an appendectomy?).

*Semantic annotation* In order to analyze text content related to a given query, Doc’EDS relies on the ECMT tool [22]. ECMT is an automatic semantic annotation program that identifies terminologies and ontology (from the HeTOP server) concepts in unstructured texts. ECMT relies on the “bag-of-words” algorithm and also on pattern-matching designed for discharge summaries, procedure reports or laboratory results which contain symbolic data (presence or absence), and numerical data. Doc’EDS embeds ECMT to analyze corpora after performing a query as a text mining tool. Therefore, it is possible to identify frequent concepts in a specific corpus (e.g. the most related diseases or the most prescribed drugs).

F. Formal evaluation of tags and segmentation

**Fig. 5:**
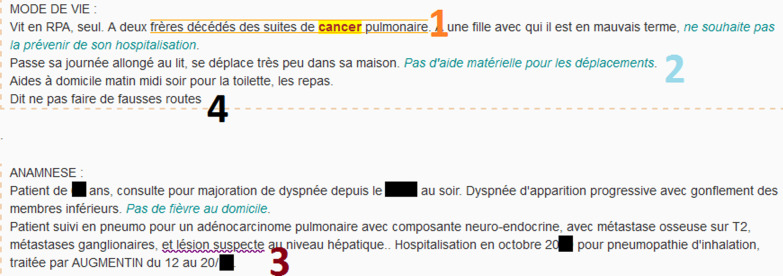
Example of Doc’EDS automatic analysis. The system highlights when it detects special contents (negations, hypotheses, family history or segments); 1/Family history (“brothers deceased after lung cancer”), 2/Negations (“no material assistance for locomotion”), 3/Hypothesis (“suspect lesion”) and 4/example of a false negative because the system failed to identify a negation (“no dysphagia reported”)

The aim of this first formal evaluation was to compute:

- The precision (TP/(FP + TP)), the recall (TP/(FN + TP)), and the F-measure (TP/(TP + 1/2(FP + FN))) of each tag (negation, hypothesis, and family medical history). Negative predictive value (TN/(TN + FN)) and specificity (TN/(TN + FP)) were also computed.
- True positive (TP) percentage of segmentation
- Negative content, family medical history content and hypothetical content occurrence among clinical concepts and documents with their 95% confidence interval.
- Random documents were drawn from hospitalization reports, consultation and procedure reports until obtaining sufficient tags (arbitrarily 250 for each category) according to the estimated ratio of this document type based on structured data. This means one hospitalization report for two procedure reports or consultations. Due to the rarity of “family medical history” tags, random reading was interrupted earlier.
- Clinical concepts from documents were manually extracted and analyzed by two public health residents as reference (TPL and PB). These two readers navigated through the randomly selected documents. The document content is displayed on the right side of the screen and corresponds to the transformed text. Words from queries are highlighted, irrelevant content is shaded, segments and tags are visible. This allows the reader to quickly evaluate relevance. The Fig. 5 illustrates how Doc’EDS is highlighting these segments and tags. In this example, a false negative of “negation tag” is shown (4), the text is not in blue italic because the system failed to detect the negation (“Dit ne pas faire de fausses routes”).

### Special content tagging

Unstructured data from narrative clinical reports are tagged with three different types of “tags”:

- “Negation” corresponds to absence (e.g. *no complication*), negative results from complementary exams (e.g. *Koch research: negative*), reject of a diagnosis (e.g. *endocarditis was excluded*) and also negative sentences (e.g. *patient is not treated by …*).
- “Family medical history” corresponds to all clinical narratives related to one or more family members. (e.g. *her father had a myocardial infarction*, *familial diabetes*).
- “Hypothesis/future” corresponds to events or facts that have not happened yet (e.g. future laboratory analyses or treatment procedures not yet scheduled), but could happen, doubts, different hypotheses of patient disease, prevention (e.g. *HBPM prevention of thrombosis*).

These three tag types are detected using regular expressions based on common nouns/verbs/adverb forms used in French. “Stop characters” are used to limit greedy expressions (e.g. dots, commas or specific words).

In the formal evaluation, tags were categorized in four modalities for each relevant clinical concept collected:

- Inappropriate tag (FP: false positive). e.g. “*Prealbumin: *0.16” Prealbumin was tagged as negative content.
- Inappropriate missing tags (FN: false negative). e.g. “*No left* heart failure”; if heart failure was not tagged as negative content it was considered as FN.
- Appropriate tags (TP: true positive)
- Appropriate missing tags (TN: true negative).

### Segmentation

Unstructured narrative clinical reports can be segmented in a maximum of 19 different structured segments (based on consensus of four clinicians and based on Rouen CDW documents): indication for hospitalization (e.g. *dyspnea and fever*), medical history, allergies, usual treatments, anamneses, clinical examinations, laboratory and imaging results, disease evolution during hospitalization, medical diagnosis, treatments received during hospitalization, prescribed treatments after hospitalization, recommendations at discharge, procedures/technical procedures (e.g. *surgical procedure*), post-operative care, geriatric assessment, post-transplantation evolution and monitoring. Segmentation is processed using regular expressions which manage lexical variants. Using segmentation in queries can be helpful because it focuses on specific discourses (e.g. searching documents containing *fall* but only in indication for hospitalization, which excludes medical history of falls)*.*

None of the 19 different categories was evaluated individually but each one was considered as a single category (contrary to the negation tag, the hypothesis tag and the family medical history tag); thus a concept was well segmented (in the correct category) or not well segmented (in wrong category or outside a category). For one same concept, a false positive segment “indication for hospitalization” could be a false negative segment “medical history” hence recall and precision are not computable. Segmentation evaluation was based on hospitalization reports only because consultation reports cannot be segmented.

An example of the evaluation data frame is provided in Table 1.

**Table 1:** Example of the data frame for formal evaluation based on extraction of manual clinical concepts (TN: true negative, TP: true positive, FP: false positive, FN: false negative, Y: yes, N: no)

Concepts	Negation	Hypothesis	Family Medical history	Well segmented
Left heart failure	TN	TN	FP	Y
Atrial fibrillation	TN	TN	TN	Y
Atrial fibrillation anticoagulant	TN	FN	TN	Y
Known allergy	TP	TN	TN	Y
« Sudden» dyspnea	TN	TN	TN	N
Lung cancer	TN	TN	TP	Y
hematemesis	TN	TP	TN	Y
narcolepsy	TP	TP	TN	Y
asthma	TP	TN	TP	Y
Prealbumin: 0.16	FP	TN	TN	Y

As the readers did not use Doc’EDS queries in these phases, they did not receive training to use these features. However, they received training for manual concept extraction, tag definition (FP, FN, TP, TN) and segmentation on five random documents. Tagging had to be exactly matched with the medical concepts to be considered as a TP, otherwise it impacted on queries (FN or FP). Reader concordance (Kappa) for each tag and segmentation was computed from document subgroups. Disagreements were resolved by consensus between the two readers as it is not necessary to add an expert since this process is only based on the understanding of the French language (e.g. is it a negative formulation or not?). There was no computation of reader concordance on extracted concepts.

G. Use cases

### Access rules

Doc’EDS can only be accessed by our team of experts and developers. Moreover, even if documents are de-identified, each document is linked to a unique patient number in the CDW. Currently, this data warehouse is composed of two distinct databases on two different servers. Thus, nominative data are stored in one small encrypted database and clinical data are stored on the other database. This type of architecture is compliant with GDPR (General Data Protection Regulation) application rules since it explicitly (physically) separates nominative data from de-identified data. Nevertheless, it is possible to re-identify patient numbers thanks to a complex decryption mechanism protected by a password only known by our team of experts. Doc’EDS cannot be accessed by the medical community. Unlike most tools based on CDWs, we believe that building queries into such complex databases with subtle algorithms is an expert task. Each use case is a dialog between the researcher and our team of experts; it often includes the assistance of a statistician methodologist to ensure that the research question can be met with the expected data extracted from Doc’EDS.

### Doc’EDS complementarity with the French hospital discharge database

In French hospitals, data retrieval is usually performed using the French hospital discharge database (Programme de médicalisation des systèmes d’information (PMSI)). Patients are identified using the CIM-10 (ICD-10 French version) and/or CCAM (for procedures). Queries are limited by the lack of codes (e.g., new practices or rare disease), the use of an inappropriate code [23, 24], absence of code due to no financial valuation (e.g. medical history), or even code evolution [25]. With a CDW, data retrieval could be optimised. We aim to show the superiority of data retrieval using Doc’EDS in two use cases.

## Results

A. Doc’EDS

Doc’EDS is a web application, written in Java EE and running on a Tomcat web container. It relies on a Lucene index and includes several additional tools that help to visualize, analyse and export results. The ETL program uses the Rouen CDW SQL database to automatically feed Doc’EDS each week from the Clinical Information System. In November 2020, data volumes were: 2 million patients, 18,123,341 narrative documents for approximately 15 million consultations/hospital stays. Doc’EDS is used in routine practice at our university hospital. So far, it has helped to respond to 140 various use cases in different specialties.

B. Tag and segmentation evaluation

A total of 5,277 (non unique) concepts were collected by the two readers among 54 hospitalization reports (3,767 concepts) and 93 procedure reports or consultations. There were 35.9 (mean) (SD = 38.8) concepts per document. Negative concepts represented 11.7% [11%; 12.6%], 4.6% [4.1; 5.2] were hypotheses, and 0.3% [0.2; 0.5] were family medical history.

In order to assess the concordance between the two readers, a subset randomly selected of the 147 documents was double-read. This subset contained 2000 (non unique) (1,737 for segmentation) concepts. Disagreements did not exceed 3.2% see Table 2. Most of the disagreements were derived from human misunderstanding or lack of training.

**Table 2:** Concordance results between the two readers (TN: true negative, TP: true positive, FP: false positive, FN: false negative)

	FN	FP	TN	TP	Kappa (CI 95%)
*Negation tag*					
FN	14	0	7	0	0.88 [0.84; 0.91]
FP	0	14	6	2	
TN	14	5	1711	4	
TP	2	7	10	204	
*Hypothesis tag*					
FN	14	0	23	2	0.70 [0.62; 0.77]
FP	0	15	0	3	
TN	19	0	1886	0	
TP	0	1	4	33	

No violation of de-identification was found among the 147 documents (0% [0; 2.5]). Concerning the negation tag and the hypothesis tag, the F measures were respectively 0.89 and 0.57 (see Table 3). Concerning segmentation (evaluation among 3767 concepts) 84% CI 95% [83%; 85%] of concepts were well segmented and concordance between readers was 0.87 [0.83; 0.90].

**Table 3:** Evaluation of (a) negation tags, and (b) hypothesis tags, resident versus Doc’EDS

Negative concepts	Reader +	Reader −	
Doc’EDS +	TP = 551	FP = 60	Precision = 0.90 [0.87; 0.92]
Doc’EDS −	FN = 68	TN = 4.598	NPV = 0.98
	Recall = 0.89 [0.86; 0.91]	Specificity = 0.98	F = 0.89

Hypothesis concepts	Reader +	Reader −	
Doc’EDS +	TP = 116	FP = 41	Precision = 0.73 [0.66; 0.80]
Doc’EDS −	FN = 128	TN = 4.992	NPV = 0.98
	Recall = 0.47 [0.41; 0.54]	Specificity = 0.98	F = 0.57

III. Use cases

Among the 140 use cases processed between January 2019 and December 2020, we chose to focus on two use cases to illustrate the complementarity between Doc’EDS and the French DRG.

### Use case 1: Transcatheter aortic valve replacement (TAVR)

TAVR is a relatively new procedure, so the corresponding codes were implemented between 2005 and 2009 in the French coding system (CCAM). Doc’EDS allowed physicians/researchers to retrieve data on endocarditis associated with TAVR procedure that could not be retrieved with DRG. Doc’EDS found 23/53 patients that DRG did not find with 2 false positives (undetected hypotheses) and 2 false negatives (documents did not mention TAVR explicitly).

### Use case 2: Fahr disease

Fahr disease or IBGC (Idiopathic Basal Ganglia Calcification) is coded with ICD-10 code G23.8 (Other specified degenerative diseases of basal ganglia). This code groups different diseases, hence it is not specific. Doc’EDS allowed physicians/researchers to refine their search in a restrained sample. The initial ICD-10 query obtained 392 patients with G23.8 and Doc’EDS found 93 patients (3 false positives). This meant that physicians/researchers did not have to read all 392 patient records to confirm Fahr disease or not.

Doc’EDS user feedback: most of the physicians/researchers who used Doc’EDS to retrieve data were satisfied by its accuracy. For example, several users reported: “this tool is a goldmine, it allowed me to find the patients that fit my study” or “thanks to Doc’EDS, I gained 3 months of hard work looking for a needle in a haystack”.

## Discussion

Doc’EDS was created in order to obtain a multilevel search engine combining structured data, clinical narratives and segmentations. Clinical narrative processing is based on a semantic approach to deal with language subtleties and enable query expansion. Doc’EDS provides basic analytics, including descriptive statistics to refine query or estimate study feasibility. The interface enables users to build rapid successive queries without having any background in information retrieval or computer science.

### Formal evaluation

Contrary to EMERSE or IT solution from Leon Berard center [26], Doc’EDS (as Dr Warehouse or as CREATE) handles negation and family medical history to deal with such subtleties. According to the tools cited above, Doc’EDS is the only one that provides a hypothesis tag in French medical documents, which represents 4.6% of concepts in our evaluation. As far as we know, previous tools did not estimate the prevalence of negative concepts (11.7%), or family medical history (< 0.5%) concepts in French medical documents.

The segmentation provided by Doc’EDS shows that over 80% of clinical concepts are well segmented. Even in hospitalization reports, segmentation was not possible because of a lack of key words used by clinicians or line breaks leading to no segmented concepts. Misplaced or wrong keywords trigger inappropriate segmentation. The 19 different segments were not evaluated or presented in other articles (just barely mentioned).

Few family medical history tags were collected; indeed, this depends on medical speciality such as oncology, pediatrics or genetics where it may be more prevalent. Even if there were only 147 documents, the external validity was high. Indeed, there are numerous and various types of clinical concepts such as clinical examination, surgical procedure, treatment, laboratory results, etc. and a wide range of expressions from different physicians/researchers. A high F-measure was registered for the negation tag (0.89) whereas the hypothesis tag obtained a low F-measure (0.57). Most of the time, the hypothesis tag weakness was derived from false negative clinical concepts corresponding to exit order (e.g. patient will have radiologic examination). Thus, it did not impact the precision of document retrieval. Moreover, there are so many variations of grammatical forms (specially in French), that we can not anticipate all forms and ways that physicians are putting their hypotheses; corresponding regular expressions will be managed progressively.

### Search mechanisms

The direct consequence of the NLP features detailed in this study is how end users can search documents in the system. More precisely, the objective of the NLP algorithm detailed here is to tag all the concepts; so, if a concept is considered as "true" in a first portion of text and then “negative” in a second portion, this situation is managed by the index during the search process. Given a document D and a concept C. Given the query R1: "C", then D will be found if C appears true at least once in D. Given the query R2: NEG("C"), then D will be found if C appears negative at least once in D. Given R3: "C" NOT NEG("C"), then D will be found if C appears true at least once and never negative.

## Conclusion

Doc’EDS is a search tool that is used on a daily basis at our university hospital in order to create cohorts for research. This tool exploits a massive corpus of more than 17.3 million documents and relies on semantic features to build sophisticated queries. Many other functions are proposed to analyze results or to refine query building (e.g. basic statistics on data distribution documents, automatic extraction concepts). Subtle semantic processing is used to detect negation, hypotheses/future, and family history. The Doc’EDS system, based on regular expressions, is continuously updated to enhance performances. This formal evaluation has shown good results for negation and average results for hypotheses/future. One other important aspect of Doc’EDS is its efficiency of performance; most queries are executed in less than two seconds. This is useful when many tests are required to obtain a “good” query.

## Perspectives

Doc’EDS provides a set of semantic functions that enable query enhancement (synonyms and related terms additions) and concept analysis to obtain an overview of relevant concepts for a given corpus. As Doc’EDS is based on the HeTOP multi-terminogy server, it contains the largest French medical lexicon. This is helpful to build queries dealing with semantic features as complex keywords (e.g. with multiple synonyms). The usefulness and usability of Doc’EDS will be the object of a future work, integrating a survey of user satisfaction and the usefulness of the tool. Doc’EDS will be re-evaluated on the 5277 clinical concepts manually extracted to increase the precision of the negation and hypothesis tags after corrections. The amount of unstructured data will grow (nurse transmissions, virology/microbiology reports, etc.). Automatic extraction is being developed around use cases to facilitate data collection for clinical research. A specific module will be developed to help researchers to build cohorts from Doc’EDS results.

## Data Availability

The datasets generated and/or analyzed during the current study are not publicly available due the GDPR application in France about individual’s data.

## Abbreviations

CDW: Clinical data warehouse
DRG: Diagnosis related groups

## Footnotes

1 https://tika.apache.org/.
