# Correction to: Evaluation of Doc’EDS: a French semantic search tool to query health documents from a clinical data warehouse

**DOI:** 10.1186/s12911-022-01839-0

**Published:** 2022-04-22

**Authors:** Thibaut Pressat-Laffouilhère, Pierre Balayé, Badisse Dahamna, Romain Lelong, Kévin Billey, Stéfan J. Darmoni, Julien Grosjean

**Affiliations:** 1 Department of Biomedical Informatics, Rouen University Hospital, Normandy, France; 2 LIMICS U1142 INSERM, Sorbonne Université & Sorbonne Paris Nord, Paris, France; 3 LITIS EA4108, Rouen University, Normandy, France

## Correction to: BMC Medical Informatics and Decision Making (2022) 22:34 10.1186/s12911-022-01762-4

Following publication of the original article [1], it was reported that the correct Fig. 1 was missing. The originally published Fig. 1 should be Fig. 4, and the originally published Fig. 4 should have been published as Fig. 5.

**Fig. 1:**
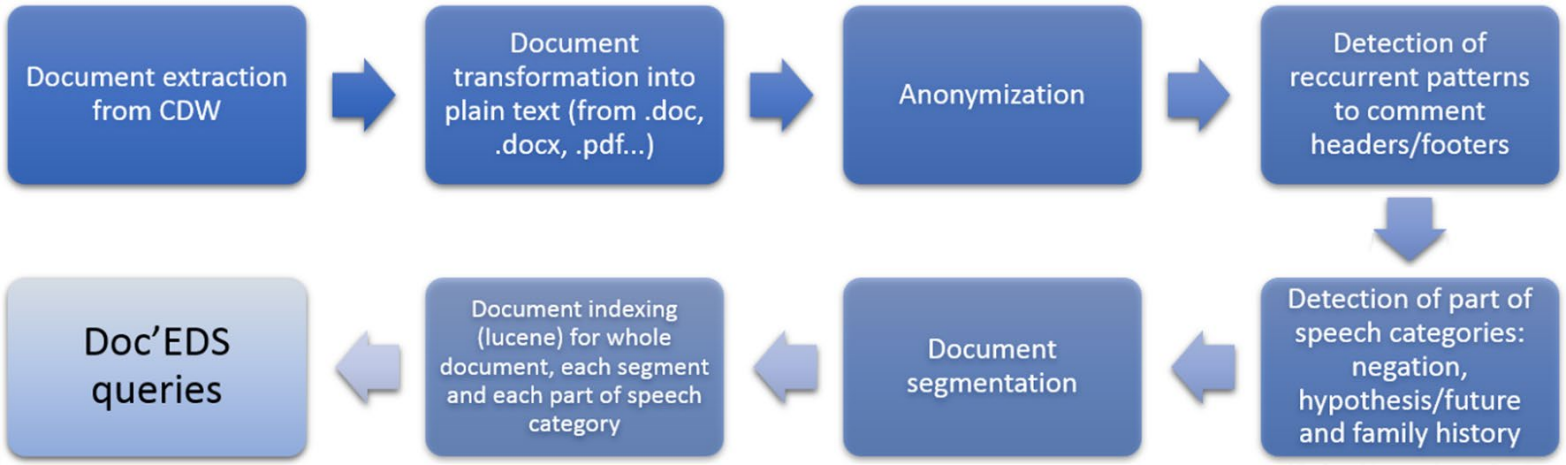
Document processing workflow from extraction to indexing

The correct Fig. 1 is provided in this Correction article. The original article [1] has been updated to include the correct Figs. 1, 4 and 5 and their corresponding in-text citations.
